# Comparison of Therapeutic Response and Clinical Outcome between HCV Patients with Normal and Abnormal Alanine Transaminase Levels

**DOI:** 10.1371/journal.pone.0142378

**Published:** 2016-03-11

**Authors:** Cheng-Kung Wu, Kuo-Chin Chang, Po-Lin Tseng, Sheng-Nan Lu, Chien-Hung Chen, Jing-Houng Wang, Chuan-Mo Lee, Ming-Tsung Lin, Yi-Hao Yen, Chao-Hung Hung, Tsung-Hui Hu

**Affiliations:** Division of Hepato-Gastroenterology, Department of Internal Medicine, Kaohsiung Chang Gung Memorial Hospital and Chang Gung University College of Medicine, Kaohsiung, Taiwan; Chiba University, Graduate School of Medicine, JAPAN

## Abstract

**Background and Aims:**

Patients with chronic hepatitic C (HCV) infection and normal serum alanine transaminase (ALT) levels were considered to have mild disease. In Taiwan, these patients were not suggested for interferon (IFN) based therapies. The aim of study is to compare therapeutic outcomes between HCV patients with normal and elevated ALT levels.

**Methods:**

We conducted a retrospective study on 3241 HCV patients treated by IFN based therapies. Patients with normal ALT levels were classified as group A (n = 186) while those with elevated ALT levels were group B (n = 3055).

**Results:**

At baseline, incidence of diabetes mellitus, low platelet counts and cirrhosis were significantly higher in group B patients. The sustained virologic response (SVR) rate was comparable between the 2 groups (65.3% vs. 65.3%, P = .993). But significantly higher incidence of HCC development after HCV treatment was observed in group B (7.4% vs. 3.2%, P = .032). No significant differences with respect to the outcome of liver decompensation, spontaneous bacterial peritonitis, and mortality were noted between 2 groups. Multivariate analysis showed younger age, female gender, non-HCV genotype 1, lower viral load, higher platelet counts and non-cirrhosis were favorable factors for achieving SVR, rather than ALT levels. Further analysis revealed older age, cirrhosis, lower platelet levels and non- peg-interferon treatment are risk factors of HCC development.

**Conclusions:**

HCV patients with normal ALT levels had similar response to antiviral therapy and low rate of HCC development after therapy. Antiviral therapies begun at early course of HCV infection may be beneficial to prevent disease progression.

## Introduction

Alanine aminotransferase (ALT) is frequently used to monitor liver injury in chronic hepatitis C (CHC)-infected patients. Up to 30%~40% of CHC-infected patients have persistently normal ALT levels (PNALT) [1–2] and are formerly referred to healthy or asymptomatic “carriers” [3]. In Taiwan, these patients were not allowed to receive interferon (IFN)-based therapies according to the criteria set by Bureau of National Health Insurance (BNHI). However, ALT levels correlated poorly with histological hepatic injury [4–5]. A significant number of CHC patients with PNALT levels have moderate to severe fibrosis, cirrhosis [6–7] and even development of hepatocellular carcinoma [8]. Disease progression can still occur [9].

Interferon-α (IFN-α) plus ribavirin for CHC and results in 40~65% sustaind virologic response (SVR) in treated patients [10–11]. Pegylated IFN plus ribavirin has been proved to increase the proportion of patients with SVR compared with interferon plus ribavirin [12]. The therapeutic response and clinical outcome of HCV patient with PNALT levels receiving anti-viral therapy was seldom discussed. Previous study from Taiwan showed that SVR rate was similar between HCV patients with PNALT levels and abnormal liver functions (67.4% vs. 65.2%) [13]. But the case number was relatively small and only IFN was used because pegylated IFN wasn’t available in Taiwan at that time. We therefore conducted the present large-scale longitudinal study on IFN-naïve CHC patients who received either IFN or Pegylated IFN combination therapy, and tried to identify the difference of therapeutic response and clinical outcome.

## Patients and Methods

From February 1999 to January 2012, 3241 CHC patients who received IFN-based (IFN or Pegylated IFN) combination therapy were enrolled. Those who had PNALT were classified as group A (n = 186) while those who had elevated ALT (> 1xULN, as 40 IU/L) were classified as group B (n = 3055). PNALT was defined as three consecutive normal ALT levels (≤1x ULN), 2 months apart over a 6-month period [14–15]. All patients were positive for anti-HCV antibodies (Ax SYM HCV 3.0; Abbott Laboratories, Chicago, IL, USA) and had detectable HCV RNA (AmplicorTM; Roche Diagnostics, Branchburg, NJ, USA). None of the patients had history of hepatic encephalopathy, hemorrhage from esophageal varices or ascites. The diagnosis of cirrhosis was made according to clinical, laboratory, abdominal ultrasonographic (US) and/or histological findings [16]. Patients with concomitant HBV or HIV infection, alcoholism or autoimmune hepatitis were excluded. Before therapy, none of these patients had HCC or suspicious space-occupying lesions as detected by ultrasound and/or CT. Before treatment, informed consent was obtained from each patient and the study was carried out in accordance with the provisions of the Declaration of Helsinki. Patients were treated with interferon, peginterferon-a-2a (Pegasys; Roche, Basel, Switzerland) at 135 or 180 mg/week subcutaneously or peginterferon-a-2b (Peg-Intron; Schering-Plough Corporation, Kenilworth, NJ, USA) at 1.5 mg/kg/week subcutaneously and oral ribavirin 800–1200 mg daily. The duration of HCC follow-up was defined from the date 24 weeks post-treatment to the date of HCC development, or to the date of last follow-up. Patients were followed up every other week in the first month, every 4 weeks subsequently until the end of therapy and every 12 weeks thereafter. SVR was defined as undetectable serum HCV RNA levels at 24 weeks after completion of treatment. Any new space-occupying lesions raising suspicion of malignancy that were detected at the time of the examination were examined by CT, magnetic resonance imaging (MRI) and/ or biopsy. The HCC diagnosis was determined according to the European Association for the Study of the Liver (EASL) and the American Association for the Study of Liver Disease (AASLD) guidelines [17–19], based on imaging features of nodules >2 cm with typical arterial vascularization using two dynamic imaging methods (CT and MRI). If this diagnostic method could not be applied, the diagnosis was histologically confirmed by liver biopsy [17–19]. This retrospective chart review study was approved by both the institutional review board and the ethics committee of Chang Gung Memorial Hospital, Taiwan (104–3276B) and the data were analyzed anonymously.

### HCV RNA and genotyping

Qualitative detection of HCV RNA was performed by a standardized RT–PCR assay (AmplicorTM; Roche Diagnostics), using biotinylated primers for the 5’-non-coding region. The lowest detection limit for this assay was 15 IU/mL. HCV genotyping was performed by a reverse hybridization assay (Inno-LiPATM HCV II; Innogenetics N.V., Gent, Belgium) using HCV-Amplicor products. Interleukin 28 B (*IL28B*)*-*Associated Variants, SNP rs12979860 was performed by PCR and fluorescence monitoring and classified as C/C, C/T and T/T alleles.

### Statistical analysis

Quantitative variables are expressed as means±SD. Comparisons of differences in categorical data between groups were performed using x2 analysis. Continuous variables were analyzed by the t-test where appropriate. Kaplan–Meier curves with log-rank test were generated for the cumulative incidence of HCC. The factors associated with SVR were evaluated by the multiple binary logistic regression analysis. Factors associated with HCC risk were determined by the Cox proportional hazard model. Risk factors selected as significant by simple Cox regression were further analyzed by multiple Cox regression. All tests were two tailed, and a P value of<0.05 was considered statistically significant.

## Results

### Patient characteristics

The baseline demographic, virological and clinical characteristics of the 3241 patients are summarized in Table 1. Patients with normal ALT levels were significantly female predominant when compared to those with elevated ALT levels (58.1% vs. 46.5%, p = .002). Patients with elevated ALT levels are more likely to be suffering from diabetes mellitus (15.2 vs. 7.5%, P = .004) and cirrhosis (22.3% vs. 15.8%, p = 0.47) compared with PNALT patients. Significantly lower platelet counts (16.6 ± 6.9 vs. 18.1 ± 7.9, p = .005) were observed in patients with elevated ALT levels. Patients with elevated ALT levels received significantly higher rate of Pegylated IFN combination therapy than patients with PNALT (69% vs. 60.8%, p = .019). The virological characteristics including viral titers, genotype and (*IL28B*)*-*Associated Variants were similar between 2 groups.

**Table 1:** Demographic, virological and clinical characteristics of the 3241 patients.

	Group A, N = 186	Group B, N = 3055	*P* value
Sex: female/male (% female)	108/78 (58.1)	1420/1635 (46.5)	.002
Mean age, ± SD	58.7 ± 12.4	59.0 ± 11.4	.727
Mean weight, kg ± SD	66.0 ± 49.8	65.6 ± 19.9	.917
Body mass index (Kg/m2)	24.7 ± 5.0	26.8 ± 6.5	.631
HCV RNA titer (IU/ml)	1811076 ± 5396422	1030158 ± 2475760	.131
≥ 4x105 (IU/ml), n (%)	58 (51.8)	881 (44.1)	.111
HCV genotype, n (%) ^a^			.122
Type 1	79 (45.7)	1499 (51.7)	
Non-1	94 (54.3)	1400 (48.3)	
Type 2, DM, n (%)	14 (7.5)	462 (15.2)	.004
Liver cirrhosis, n (%)	27 (15.8)	664 (22.3)	.047
Laboratory data			
ALT	29.49 ± 7.7	169.4 ± 225.9	< .001
Prothrombin time, S ± SD	7.8 ± 6.6	8.7 ± 4.8	.078
Platelet counts, 109/L ± SD	18.1 ± 7.9	16.6 ± 6.9	.005
Alpha-feto protein, ng/mL± SD	8.1 ± 55.5	14.3 ± 44.7	.070
IL-28B, n (%) ^b^			.848
CC allele	74 (87.1)	1683 (86.2)	
CT allele	11 (12.9)	263 (13.5)	
TT allele	0 (0)	7 (0.3)	
Treatment, n (%)			.019
With standard interferon	73 (39.2)	947 (31.0)	
With peg-interferon	113 (60.8)	2108 (69.0)	

Abbreviation: DM, diabetes mellitus; HCV, hepatitis c virus; ALT, alanine amonotransferase

a. 173/2899 data available

b. 85/1953 data available

### Outcomes between Patients with Normal ALT and Elevated ALT Levels (Table 2)

The sustained virologic response (SVR) rate was comparable between PNALT and elevated ALT groups (65.3% vs. 65.3%, p = 993). Significantly higher incidence of HCC was observed in group B compared with group A (7.4% vs. 3.2%, p = .032). No significant differences with respect to the outcome of decompensated cirrhosis and its complications including ascites, variceal bleeding, hepatic encephalopathy and spontaneous bacterial peritonitis were noted between the 2 groups. The mortality rate was similar (3.2% vs. 2.9%, p = .815) between 2 groups.

**Table 2:** Outcomes between Patients with PNALT and Elevated ALT Levels.

	Group A, N = 186	Group B, N = 3055	*P* value
SVR, n (%)	111 (65.3)	1788 (65.3)	.993
HCC, n (%)	6 (3.2)	225 (7.4)	.032
EV bleeding, n (%)	4 (2.2)	34 (1.1)	.205
Hepatic encephalopathy, n (%)	2 (1.1)	35 (1.2)	.925
SBP, n (%)	0 (0)	11 (0.4)	.411
Ascites, n (%)	5 (2.7)	60 (2.0)	.500
Mortality, n (%)	6 (3.2)	89 (2.9)	.815

Abbreviation: SVR, sustained virological response; HCC, hepatocellular carcinoma; EV, oesophageal varices; SBP, spontaneous bacterial peritonitis

### Univariate and Multivariate analysis of predictive factors associated with SVR

Because the data of IL-28B was not complete, it’s not enrolled for analysis. Under univariate analysis, the achievement of SVR was significantly higher in patients with younger age (<60 years old, p < .001), female gender (p = .005), lower viral loads (<4x10^5^ IU/ml, p < .001), non-genotype 1 (P < .001), higher platelet counts (≥15 x 10^9^/L, P < .001), lower AFP levels (<20 ng/ml, P < .001) and peg-IFN treatment (P = .039). Cirrhosis and diabetes mellitus were significant negative factors associated with achieving SVR under univariate analysis. After multivariate analysis, younger age, female gender, lower viral loads, higher platelet counts, non-genotype 1 and non-cirrhosis, were independent factors for achieving SVR (Table 3). There was no significant difference of SVR rate with regards to ALT level (Elevated ALT level vs. PNALT levels, Odds ratio (OR) 1.001 CI 0.723~1.387, P = .993). In patients with PNALT, genotype non-1, higher platelet, non-cirrhosis and non-DM were favorable factors to achieve SVR. On multivariate analysis, only liver cirrhosis was the significant negative factor associated with SVR (Table 3).

**Table 3:** Univariate and Multivariate analysis of factors associated with SVR.

Variable	Comparison	Univariate analysis		Multivariate analysis	
		Odds ratio (95% CI)	*P* value	Odds ratio (95% CI)	*P* value
All patients (n = 3241)					
Age (Year)	≥ 60 vs. <60	0.564 (0.482~0.659)	< 0.001	0.747 (0.594~0.941)	.013
Gender	Female vs. Male	1.243 (1.067~1.449)	0.005	1.364 (1.090~1.708)	.007
HCV RNA titer (IU/ml)	≥ 4x10^5^ vs. < 4x10^5^	0.445 (0.366~0.543)	< 0.001	0.522 (0.417~0.654)	< .001
Genotype	1 vs. Non-1	0.237 (0.200~0.282)	< 0.001	0.264 (0.208~0.334)	< .001
Platelet counts (10^9^/L)	≥ 15 vs. < 15	2.031 (1.739~2.373)	< 0.001	1.508 (1.185~1.919)	.001
Liver cirrhosis	Yes vs. No	0.252 (0.210~0.302)	< 0.001	0.357 (0.273~0.468)	< .001
Diabetes mellitus	Yes vs. No	0.739 (0.603~0.906)	0.004		NS
AFP (ng/ml)	≥ 20 vs. < 20	0.544 (0.438~0.676)	< 0.001		NS
Peg-interferon treatment	Yes vs. No	1.196 (1.009~1.418)	0.039	3.925 (1.821~8.456)	NS
ALT levels	Elevated vs. PNALT	1.001 (0.723~1.387)	0.993		NS
Group A patients (n = 186)					
Age (Year)	≥ 60 vs. <60	0.720 (0.382~1.358)	0.310		
Gender	Male vs. Female	0.529 (0.279~1.003)	0.051		NS
HCV RNA titer (IU/ml)	≥ 4x105 vs. < 4x105	0.670 (0.295~1.520)	0.337		
Genotype	1 vs. Non-1	0.410 (0.208~0.810)	0.010		NS
Platelet counts (109/L)	≥ 15 vs. < 15	2.033 (1.050~3.935)	0.035		NS
Liver cirrhosis	Yes vs. No	0.117 (0.045~0.301)	< 0.001	0.140 (0.048~0.409)	< .001
Diabetes mellitus	Yes vs. No	0.262 (0.083~0.822)	0.022		NS
AFP (ng/ml)	≥ 20 vs. < 20	0.527 (0.032~8.584)	0.653		
Peg-interferon treatment	Yes vs. No	1.413 (0.739~2.701)	0.296		

Abbreviation: AFP, Alpha-feto protein; HCV, hepatitis c virus; ALT, alanine amonotransferase; NS, not significant

### Cumulative incidence of HCC and Factors associated with the development of HCC

**Fig 1:**
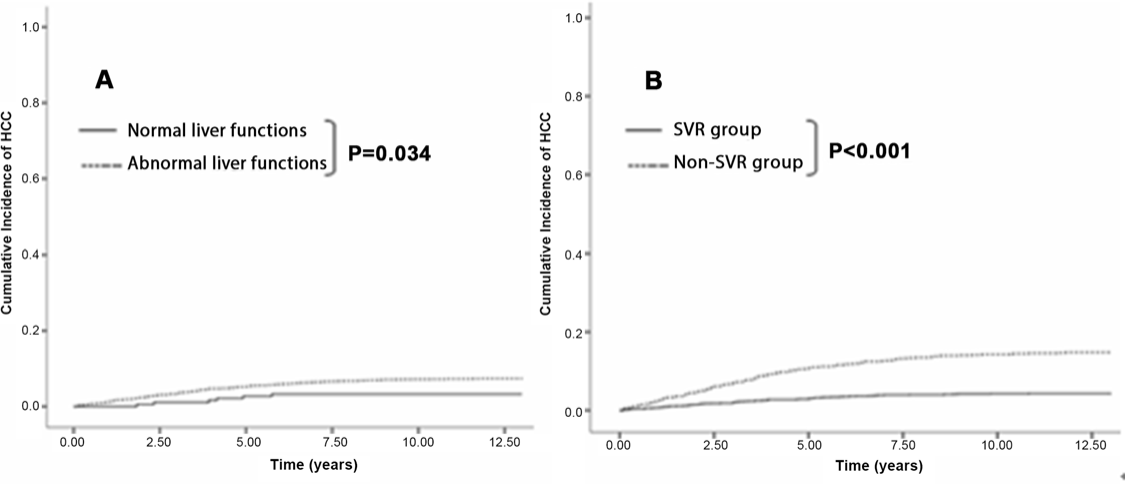
Cumulative Incidence of HCC development after IFN-based therapy. (A) Patients with abnormal liver functions had significantly higher incidence of HCC than those with normal liver functions (P = 0.034). (B) Patients who achieved sustained virological response (SVR) had significantly lower incidence of HCC than those who didn’t (P<0.001).

**Fig 2:**
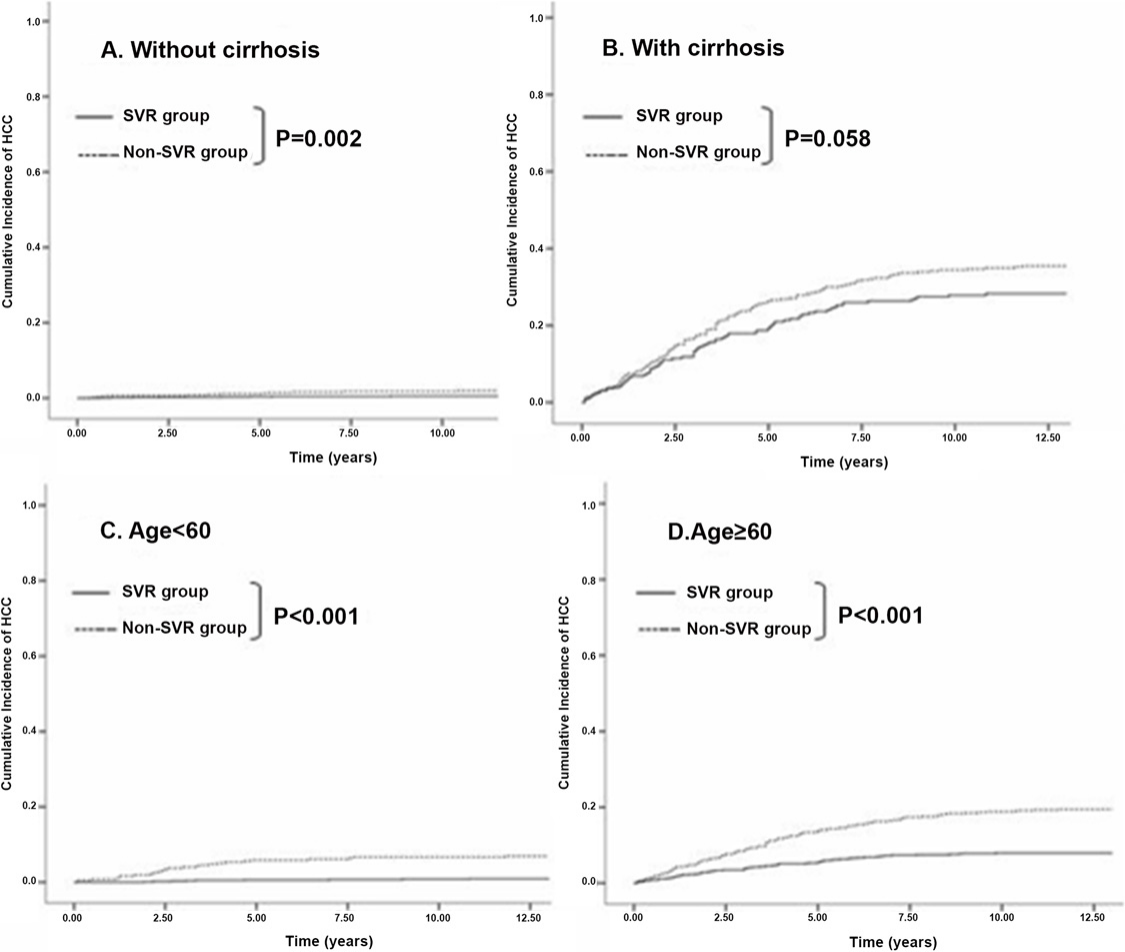
Cumulative Incidence of HCC development after IFN-based therapy. (A) For the group without cirrhosis, patients who achieved SVR had significantly lower incidence of HCC than those who didn’t (P = 0.002). (B) The trend of lower incidence of HCC in cirrhotic patients with SVR than those without SVR was observed (P = 0.058). (CD) Significantly lower incidence of HCC was observed in patients with SVR than those without SVR. It was identical when age<60 (P < .001) and age≥60 (P < .001) were concerned.

**Fig 3:**
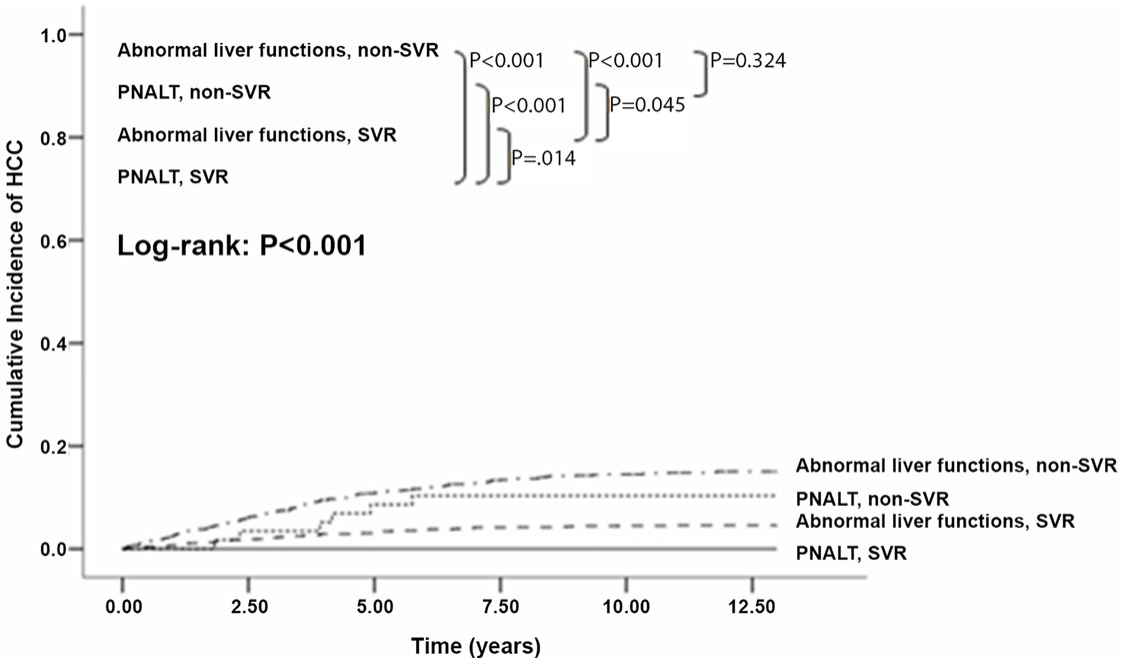
Cumulative Incidence of HCC development after IFN-based therapy according to liver function and viral eradication status. Significantly higher incidence of HCC was observed in patients with abnormal liver functions and non-SVR status, followed by PNALT group with non-SVR, abnormal liver functions with SVR and PNALT with SVR (log-rank test, P < .001).

Figs 1–3 show the cumulative incidence of HCC development using the Kaplan-Meier method and log-rank test. Patients with elevated ALT levels developed HCC at a significantly higher rate than those with PNALT levels (P = .034). Compared to patients without SVR, those who achieved SVR had significantly lower incidence of HCC (P < .001). This finding was identical when different subgroups were analyzed: in patients with no cirrhosis (P = .002), age<60 (P < .001) and age≥60 (P < .001). The trend of lower incidence of HCC in cirrhotic patients with SVR than those without SVR was also observed (P = .058). Moreover, when liver functions and viral eradication status were taken together for analysis, we found that significantly higher incidence of HCC was observed in patients with abnormal liver functions and non-SVR status, followed by PNALT group with non-SVR, abnormal liver functions with SVR and PNALT with SVR (log-rank test, P < .001).

Univariate analysis showed that older age, cirrhosis, diabetes mellitus, lower platelet counts, genotype 1, more prolonged prothrombin time and higher AFP level were risk factors of HCC development. Achievement of SVR, PNALT levels and peg-IFN treatment were significant negative factors associated with HCC development. Further multivariate analysis revealed that older age, liver cirrhosis, lower platelet levels, non-SVR and non- peg-IFN treatment are independent risk factors of HCC development (Table 4).

**Table 4:** Univariate and Multivariate analysis of risk factors associated with occurrence of HCC.

Variable	Comparison	Univariate analysis		Multivariate analysis	
		Hazard ratio (95% CI)	*P* value	Hazard ratio (95% CI)	*P* value
Age (year)	≥ 60 vs. <60	5.350 (3.749~7.634)	< 0.001	2.185 (1.518~3.147)	< .001
Gender	Male vs. Female	1.082 (0.835~1.401)	0.553		
HCV RNA titer (IU/ml)	≥ 4x10^5^ vs. < 4x10^5^	0.891 (0.645~1.232)	0.485		
Genotype	1 vs. Non-1	1.741 (1.320~2.296)	< 0.001		NS
Platelet counts (10^9^/L)	≥ 15 vs. < 15	0.183 (0.134~0.249)	< 0.001	0.691 (0.500~0.955)	.025
Liver cirrhosis	Yes vs. No	44.632 (28.208~70.620)	< 0.001	30.136 (18.172~49.979)	< .001
Diabetes mellitus	Yes vs. No	2.514 (1.894~3.336)	< 0.001		NS
Prothrombin time (sec)	Per 1 unit increase	1.028 (1.011~1.045)	0.006		NS
AFP (ng/ml)	≥ 20 vs. <20	3.668 (2.787~4.826)	< 0.001		NS
Peg-interferon treatment	Yes vs. No	0.765 (0.586~0.998)	0.049	0.689 (0.520~0.912)	.009
ALT levels	Elevated vs. PNALT	2.340 (1.040~5.263)	0.040		NS
SVR	SVR vs. Non-SVR	0.280 (0.214~0.367)	< 0.001	0.718 (0.541~0.952)	.021

Abbreviation: AFP, Alpha-feto protein; SVR, sustained virological response; HCV, hepatitis c virus; ALT, alanine amonotransferase; NS, not significant

## Discussion

Previous studies showed 30~40% of patients with HCV infection have PNALT levels [1–2,20]. The degree of liver damage was formerly considered to be minimal or mild [14,21]. However, recent studies had shown that these patients had some degree of liver damage on biopsy and were even at risk of developing HCC. The natural history of HCV carriers with PNALT was not always benign.

Recent studies revealed that about 17%~86% of patients with PNALT levels had ALT flare-ups during follow-ups [13,22–23], and hence caused the inflammation as well as disease progression. Liver biopsy clarified the status of liver fibrosis and might be able to help to guide making the decision whether patients with PNALT levels need antiviral therapy or not. Patients with initial fibrosis F2 or F3 were more likely to develop cirrhosis and therefore should be treated [24]. However, it is not practical to perform routine biopsy on all the patients in real world because of potential for complications resulting from this procedure. Old age, male gender, initial fibrosis: F2-F3, ALT flares and overweight were predictive factors for disease progression in patients with PNALT levels [8, 24–26]. Among them, age is important for decision making. As patients age, they were suffering from longer durations of HCV infection, which in turn would increase the severity of the liver fibrosis [27]. Moreover, younger patients had less co-morbidity and would tolerate the therapy better than elder patients. Previous studies also showed that those who achieved SVR after antiviral therapy had better quality of life, less fatigue and even increased life expectancy [28–29]. Apart from this, HCV carriers may experience extrahepatic manifestations including lymphoma, mixed cryglobulinemia and multiple autoimmune diseases, which in times might be more severe than HCV infection itself. Thus, optimal antiviral therapy should be started as early as possible when patients get HCV infection, especially for younger group in the presence of predictive factors for SVR described above.

This current study showed that SVR rate was identical between PNALT and elevated ALT groups (65.3% vs. 65.3%, p = 993). Patient-related factor, including older age and male gender; virus-related factors, including genotype 1, higher viral loads; and disease-related factors such as cirrhosis and lower platelet counts were observed to be risk factors of non-SVR among all HCV patients in this study. But the alanine aminotransferase level, either normal or elevated, was not associated with SVR rate. The similar response to antiviral therapy between patients with normal and abnormal liver functions was well established [30–31]. But the comparison of clinical outcomes between of 2 groups was seldom discussed. In this present study, we found that patients who achieved SVR had significantly lower incidence of HCC development compared to those who didn’t (P < .0001). Besides, those with PNALT levels were found to have lower incidence of HCC development compared to those with elevated ALT levels (P = .034). The factors including older age, liver cirrhosis, lower platelet levels, non-SVR and non- peg-IFN treatment were analyzed to be risk factors associated with development of HCC, rather than the alanine aminotransferase level. Among them, the presence of cirrhosis was the strongest risk factor. This was reasonable because cirrhosis reflected the later stage of liver damage and hence carried greater risks of HCC development. When liver functions and viral eradication status were taken together for analysis, we found that PNALT patients with non-SVR status had significantly higher rate of HCC development than those with abnormal liver functions and SVR status (Fig 3, P = .045). The clinical course in patients with HCV and PNALT is not always benign.

Our theory is, since the ALT activity doesn’t not affect the response to combination therapy, the timing of treatment should therefore be focused on liver damage, age, viral factors, co-morbidities and patient’s willingness, rather than a single biochemical test. As for the cost-benefit ratio, the morbidity and mortality could be reduced if patients with PNALT level were treated at the same rate as those with elevated ALT levels [32]. The era of direct-acting antiviral (DAA) therapy has come and promises “IFN-free” therapy and also has shown much better SVR rate than IFN-based therapies. The percentage of patients falling into the non-SVR category will likely be quite low. However, analysis of the present study will impact clinical care moving forward on the SVR patients in interferon era. The concept became eradication and thus all HCV patients should receive antiviral treatment, either with normal or abnormal liver functions. However, DAA therapy is quite expensive and not supported by National Health Insurance in every country. In the country whose first line treatment is interferon-based therapy, the result of this study provides an useful information for clinical guidance.

There were some limitations in this study. The first was the heterogeneity of our antiviral regimens, which included IFN or pegylated interferon combination therapy. Second, not all patients enrolled in this study received liver biopsy before or after treatment because some patients were concerned about potential complications. Besides, in Taiwan, HCV-infected patients with PNALT were not allowed to receive IFN-based therapies according to the criteria set by Bureau of National Health Insurance (BNHI). We were only able to enrolled patients who were willing to receive antiviral therapy at his/her expense without national health insurance coverage. As a result, the portion of patients with PNALT was smaller compared to the whole sample population.

## Conclusion

In the current study, HCV patients with normal ALT levels had similar response to antiviral therapy and low rate of HCC development after therapy. The clinical course is not always benign. Antiviral therapies begun at early course of HCV infection may be beneficial to prevent disease progression.

## Supporting Information

S1 Dataset Minimal data of HCV patients receiving antiviral therapies in our program. (XLS)
